# Transcriptomics reveals a distinct metabolic profile in T cells from severe allergic asthmatic patients

**DOI:** 10.3389/falgy.2023.1129248

**Published:** 2023-05-31

**Authors:** Carmela Pablo-Torres, Carlota Garcia-Escribano, Martina Romeo, Cristina Gomez-Casado, Ricardo Arroyo Solera, José Luis Bueno-Cabrera, M. del Mar Reaño Martos, Alfredo Iglesias-Cadarso, Carlos Tarín, Ioana Agache, Tomás Chivato, Domingo Barber, María M. Escribese, Elena Izquierdo

**Affiliations:** ^1^ Institute of Applied Molecular Medicine Instituto de Medicina Molecular Aplicada Nemesio Díez (IMMA), Department of Basic Medical Sciences, Facultad de Medicina, Universidad San Pablo-CEU, CEU Universities, Urbanización Montepríncipe, Madrid, Spain; ^2^ Department of Hematology, Hospital Universitario Puerta de Hierro Majadahonda, Madrid, Spain; ^3^ Department of Allergy, Hospital Universitario Puerta de Hierro Majadahonda, Madrid, Spain; ^4^ R+D Department, Atrys Health, Madrid, Spain; ^5^ Faculty of Medicine, Transylvania University, Brasov, Romania

**Keywords:** t cells, CD3+cells, allergy, transcriptomics, metabolism, severe phenotype, inflammation, Tregs

## Abstract

The reasons behind the onset and continuation of chronic inflammation in individuals with severe allergies are still not understood. Earlier findings indicated that there is a connection between severe allergic inflammation, systemic metabolic alterations and impairment of regulatory functions. Here, we aimed to identify transcriptomic alterations in T cells associated with the degree of severity in allergic asthmatic patients. T cells were isolated from severe (*n* = 7) and mild (*n* = 9) allergic asthmatic patients, and control (non-allergic, non-asthmatic healthy) subjects (*n* = 8) to perform RNA analysis by Affymetrix gene expression. Compromised biological pathways in the severe phenotype were identified using significant transcripts. T cells' transcriptome of severe allergic asthmatic patients was distinct from that of mild and control subjects. A higher count of differentially expressed genes (DEGs) was observed in the group of individuals with severe allergic asthma vs. control (4,924 genes) and vs. mild (4,232 genes) groups. Mild group also had 1,102 DEGs vs. controls. Pathway analysis revealed alterations in metabolism and immune response in the severe phenotype. Severe allergic asthmatic patients presented downregulation in genes related to oxidative phosphorylation, fatty acid oxidation and glycolysis together with increased expression of genes coding inflammatory cytokines (e.g. IL-19, IL-23A and IL-31). Moreover, the downregulation of genes involved in TGF*β* pathway together with a decreased tendency on the percentage of T regulatory cell (CD4 + CD25+), suggest a compromised regulatory function in severe allergic asthmatic patients. This study demonstrates a transcriptional downregulation of metabolic and cell signalling pathways in T cells of severe allergic asthmatic patients associated with diminished regulatory T cell function. These findings support a link between energy metabolism of T cells and allergic asthmatic inflammation.

## Introduction

Severe allergic asthmatic patients exhibit a chronic inflammatory status with frequent exacerbations that lead to irreversible tissue damage and epithelial barrier dysfunction (1, 2). Despite receiving high doses of corticosteroids, immunotherapy, or even biological drugs, these patients remain unresponsive, leading to a poor quality of life. They also experience several comorbidities, which result in numerous hospital admissions over the years (1, 3–5).

We have previously shown that severe respiratory allergic patients have specific platelet content, reduced protein synthesis, and switch of immune cells' metabolism to aerobic glycolysis (6, 7). It has been widely described how systemic metabolism reprograms the fate of immune cells, by influencing their activation, proliferative capacity and their quiescent state in tissues and in the systemic circulation (8). Interestingly, emerging evidence indicates that immunosuppressive drugs target T-cell metabolism and metabolic checkpoints, which could contribute to their immunosuppressive effects (9). In a similar way, metabolic modifications of severe allergic patients might also affect T cell functions. Nonetheless, the particular modifications in T cells and their contribution to exacerbated allergic responses are still not well understood. Our previous proteomic data of serum samples points to the activation of T cells in uncontrolled asthmatic patients (1). However, whether or not allergy severity is related to specific changes in T cells is a controversial issue. The transcriptomic alteration of T cells from sputum and bronchoalveolar lavage fluid (BAL) has been previously described in severe asthmatic patients (10). However, gene expression changes were not confirmed in T cells from peripheral blood (11). Nevertheless, these studies included both non-allergic and allergic asthmatic subjects in the study, a fact that could interfere with the outcome.

In this work, we aim to better understand T cell transcriptomic modifications associated to severe allergic asthma phenotypes. A better knowledge of T cell features in relation to the severity degrees of allergic diseases will shed light on the mechanisms underlying the chronic inflammatory status of severe allergic patients.

## Materials and methods

### Patients

Twenty-four individuals were recruited between October 2018 and February 2021 after the approval of the protocol by the Committees of Research and Ethics from the Hospital Universitario Puerta de Hierro Majadahonda (HUPHM). Written informed consent was obtained from all subjects. Eight non-allergic non-asthmatic healthy subjects were recruited and used as controls. A group of sixteen allergic asthmatic patients was recruited from HUPHM Allergy Service. Sensitization was defined by clinical history of allergy to aeroallergens documented by skin prick test (SPT). Allergic patients were stratified by severity in mild (*n* = 9) and severe (*n* = 7) groups according to GINA (Global Initiative for Asthma) guidelines (12). Severe patients belonged to GINA Step 5, meaning that they met at least one of the following criteria: (1) Poor asthma control assessed by ACT (Asthma Control Test) < 20 or ACQ (Asthma Control Questionnaire) > 1.5; (2) Two or more severe exacerbations/ two or more glucocorticosteroid cycles of more than three days each (along the previous year); (3) One or more hospitalizations for a severe exacerbation (in the previous year). The rest of the patients were included in the mild allergic asthma group (Table 1, Supplementary Tables S1, S2). None allergic asthmatic patient presented steroid resistance. Exclusion criteria for the study were patients under the age of 18, as well as those with co-existing inflammatory diseases, metabolic disorders, cancer, or haematological diseases.

**Table 1:** Subject's clinical characteristics.

Characteristics		Control group (*n* = 8)	Mild group (*n* = 9)	Severe group (*n* = 7)	*P*-value
		Mean (95% CI) or number of patients			
Demographics (Allergy Service HUPH)					
Gender (Female) ^a^		7	7	7	ns
Age^d^		31.13 (26.06, 36.19)	33.33 (26.46, 40.21)	35.86 (25.35, 46.37)	ns
Smoking ^a^		1	2	0	ns
Allergy onset age^b^			16.44 (9.20, 23.69)	17.29 (9.97, 24.60)	ns
Reactions (RC/RC + AS) ^a^			2/7	0/7	ns
SPT^a^	Olive		9	3	0.0192*
	Grass		9	4	0. ns
	Cupressus arizonica		6	6	ns
	Platanus		6	1	ns
	Cynodon		5	3	ns
	Weeds		6	2	ns
	Fraxinus		8	1	0.0087**
	Profilin		2	1	ns
	Alternaria		2	0	ns
	Dpt		1	4	ns
	Dfar		2	4	ns
	Cat		4	3	ns
	Dog		1	4	ns
FVC^a^ (<80% predicted)			0	5	0.0278*
FEV1^a^ (<80% predicted)			0	5	0.0278*
WBC (x10^9^/l)^d^		6.33 (5.55, 7.11)	6.96 (5.62, 8.29)	1.06 (5.13, 8.99)	0.6724

*p < 0.05; **p < 0.01.

^a^ Fisher`s exact test.

^b^ Mann-Whitney test.

^c^ Chi-square.

^d^ Kruskal-Wallis test.

CI, confidence interval; Freq, frequency; ns, non-significative; RC, rhinoconjunctivitis; AS, asthma; SPT, skin prick test; Dpt, Dermatophagoides pteronyssinus; Dfar, Dermatophagoides farinae; FVC, forced vital capacity; FEV1, first second of forced respiration; MPV, mean platelet volume; WBC, white blood cells.

### Isolation of peripheral blood mononuclear cells (PBMCs) from the leukocyte reduction system chamber (LRSC)

By plateletpheresis process, we obtained blood retained in LRSC and used it for PBMC isolation (Supplementary Figure S1). Briefly, plateletpheresis was performed in the Apheresis Unit of the Haematology department of the hospital. Trima Accel machine (Terumo BCT) was set to obtain PRP (85 ml) and platelet-poor plasma (PPP) (50 ml) samples using Adenine Citrate Dextrose-A (ACD-A) as anticoagulant. Blood contained in the LRSC was diluted in 1 V of RPMI medium (Thermo Fisher Scientific) and carefully dispensed onto 1 V of Ficoll (Thermo Fisher Scientific). After centrifugation, the PBMC fraction was collected and washed with PBS. Details of the above-mentioned protocol can be found elsewhere (13).

### CD3^+^ cell isolation and RNA extraction

T cells (CD3^+^) were isolated from the PBMC fraction with magnetic MicroBeads (Miltenyi Biotec) following manufacturer instructions for manual isolation with MS columns (Miltenyi Biotec) and MidiMACS magnets (Miltenyi Biotec). Once isolated, cell populations were stored in Rneasy Lysis (RLT) buffer containing 1% *β*-mercaptoethanol at −20°C until transcriptomic analysis. RNA was extracted from T cells using Rneasy® Mini Kit (Qiagen) with Dnase treatment following manufacturer procedure. RNA concentration and its integrity were assessed with Experion RNA StdSens analysis kit (Bio-Rad Laboratories Inc.), establishing an RNA quality indicator (RQI) ≥7 as a requisite for transcriptomic analysis.

### CD4^+^, CD4^+^ CD25^−^ and CD4^+^ CD25^+^ cell isolation

CD4^+^ cells were isolated from control (*n* = 6), mild (*n* = 3) and severe (*n* = 6) allergic patients from the PBMC fraction with magnetic MicroBeads (Miltenyi Biotec) through negative selection following manufacturer instructions for manual isolation. A fraction of the total CD4 + isolated cells was used for isolating CD4 + CD25^−^ (Teff) and CD4 + CD25^+^ (Treg) cells with magnetic MicroBeads (Miltenyi Biotec) through positive selection following manufacturer instructions. Teff and Treg were quantified using a Neubauer chamber.

### Microarray gene expression

Transcriptomic analysis of CD3^+^ T cells (*n* = 23) was performed using GeneChip Human Gene 2.1 ST strips (Affymetrix, Thermo Fisher Scientific). Following manufacturer instructions, 100 ng RNA from each sample were hybridized using GeneChip™ WT PLUS Reagent Kit. Hybridization details were previously described (6).

### Data treatment and statistics

The normalization and transformation of CEL files into expression measures were performed using R software. The process involved background correction, normalization, and summarization of the probe set-level, which was carried out using the Robust Multi-Array Average (RMA) method. The normalized intensities were used for constructing partial least squares discriminant analysis (PLS-DA) models using SIMCA P + 14.0 (Umetrics, Umeå, Sweden). The robustness of the models was evaluated by *R*^2^ (explained variance) and Q^2^ (capability of prediction) scores. Then, we performed univariant statistics using Matlab R2015a (Mathworks, Natick, Massachusetts, USA). As this is a pilot study, a gene was considered differentially expressed (DEG) with a Mann-Whitney *p*-value < 0.05 (Supplementary Table S3). Selected genes were validated by qRT-PCR (Supplementary Figure S4). The top 100-fold change DEGs were used for constructing heatmaps using MetaboAnalyst 5.0 software. Pathway analysis was performed with DEGs and non-significant transcripts as background using GOrilla software. Trajectories of DEGs related to OXPHOS, FAO, glycolysis, cytokine activity and TGF-*β* signalling pathways were plotted using Prism 8. The microarray data have been deposited in NCBI Expression Omnibus (GEO) database and are accessible through GEO Series accession number GSE224253.

## Results

### Patient classification

The clinical history of all subjects was carefully examined, and no differences related to sex, age, smoking status, or age of onset were found among the groups. (*p *> 0.05) (Table 1). All severe allergic asthmatic patients (*n* = 7) presented rhinoconjunctivitis and asthma, as well as most of mild patients (*n* = 7). Patients did not show differences regarding their sensitization profile, aside from *Olea spp*. and *Fraxinus spp*. pollen (*p *< 0.05). There were statistically significant differences in Forced Vital Capacity (FVC) and Forced Expiratory Volume in one second (FEV1) between the mild and severe groups. Most severe asthmatic patients exhibited pathological levels (<80%) of these parameters. There were no differences in white blood cell counts between the experimental groups as observed in the whole blood hemograms. Individual data of all the patients are shown on Supplementary Tables S1, S2.

### T cells from severe allergic asthmatic patients display a particular transcriptomic fingerprint

**Figure 1:**
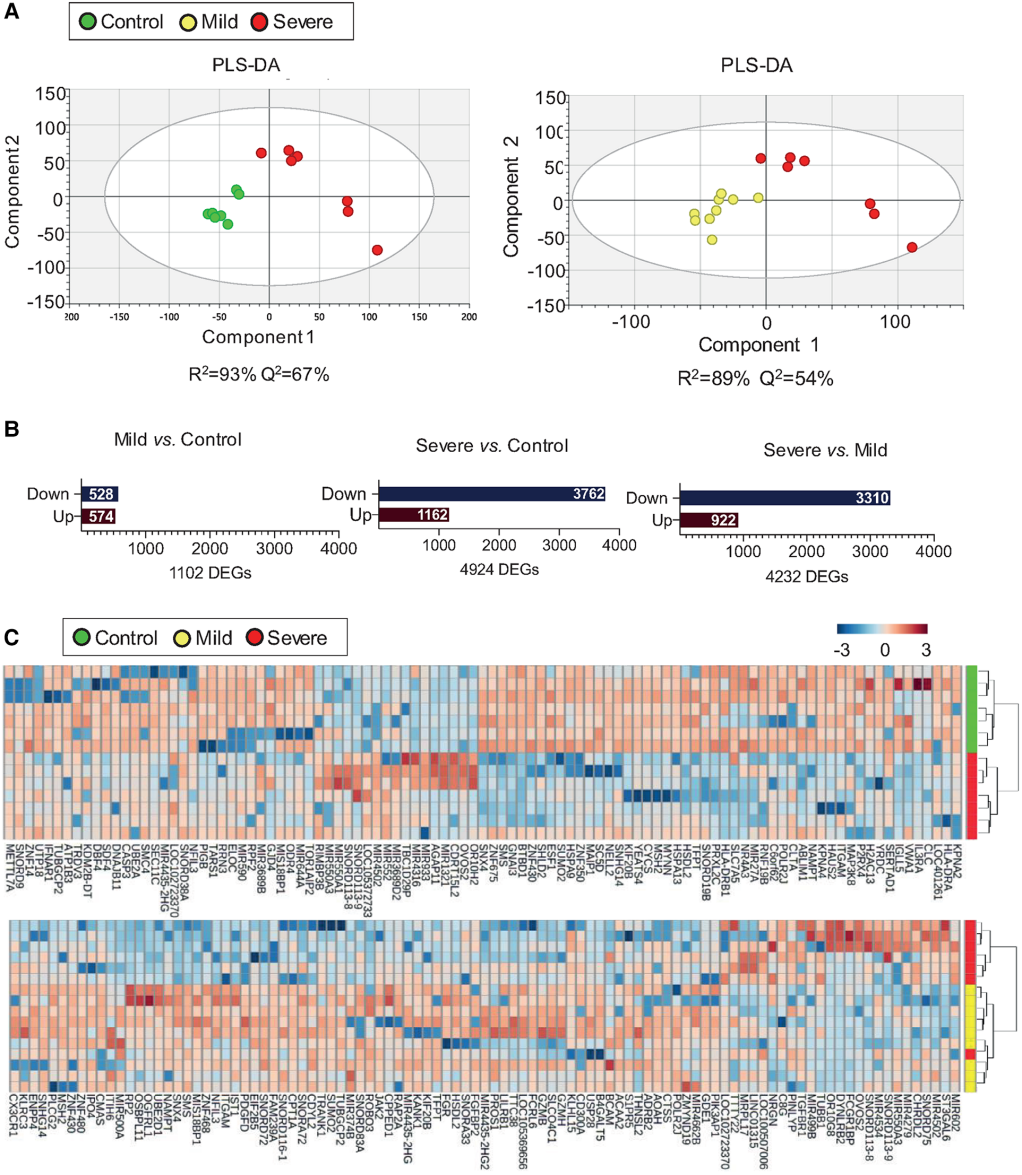
T cells from severe allergic patients display a unique transcriptomic fingerprint. (**A**) PLS-DA from T cells’ transcriptomic data showing differences between Severe (*n* = 7) vs. Control (*n* = 7) (left) and between Severe (*n* = 7) vs. Mild (*n* = 9) (right). Two components were displayed and are represented by X and Y-axis respectively. (**B**) Bar plot representation indicating the number of downregulated (Down) and upregulated (Up) DEGs (Mann-Whitney U test *p* value < 0.05) between Mild vs. Control (left), Severe vs. Control (centre) and Severe vs. Mild (right). (**C**) Hierarchical clustering was performed with the top 100-fold change DEGs between Severe vs. Control (upper) Severe vs. Mild (lower). Each row represents a single transcript; each column represents an individual T cell sample. Red bands indicate a higher expression level, and blue bands indicate a lower expression level.

Evidence support that different types of T cells contribute to allergic inflammation. The aim of the project was to gain insight into the main alterations in T cell response to later deeply study the key T cell subtypes involved in severe allergic inflammation. Therefore, we considered investigating the transcriptomic fingerprint of the whole CD3 T cells population. To identify transcriptomic changes associated to severity in T cells the T cell transcriptomic data was studied by a PLS-DA model with the three study groups (Supplementary Figure S2). A slight clustering was observed when comparing the three groups (*R*^2 ^= 34%, *Q*^2 ^= 26%), but a strong classification was observed when comparing mild or control subjects two-by-two with the severe asthmatic group (Figure 1A). T cells from severe allergic asthmatic patients displayed a transcriptomic profile that differentiates them from control (*R*^2^ = 93% and *Q*^2^ = 67%) and mild (*R*^2^ = 89% and *Q*^2 ^= 54%) subjects. Next, we explored the specific differences associated to the severe allergic asthmatic phenotype by a differential gene expression analysis (Supplementary Table S3 and Supplementary Figure S3A). The severe allergic asthmatic group showed a greater number of gene transcript alterations in comparison to the mild asthmatics (4,232 genes) and control (4,924 genes) groups. Likewise, we could find differences when comparing mild *vs.* control group (1,102 genes), which confirmed disease-related gene alterations on CD3-positive cells, although results showed a lower clustering of the subjects than in the comparison with the severe group (Figure 1B and Supplementary Figure S3A,B). Of note, the number of downregulated DEGs was considerably higher than upregulated DEGs in the severe allergic asthmatic group when comparing with mild allergic asthmatic group (3,310 downregulated genes) and control (3,762 downregulated genes) subjects (Figure 1B). Next, we performed hierarchical clustering with the top 100 DEGs in the pairwise comparisons of severe patients (Figure 1C). Results showed that severe allergic asthmatic patients were clustered when comparing to mild and control subjects, particularly in the latter case. Therefore, T cells from severe allergic asthmatic patients present a characteristically downregulated transcriptomic signature compared to the other groups.

### Metabolic transcriptome signature of T cells from severe allergic asthmatic patients shows a decrease in oxidative phosphorylation, fatty acid oxidation and glycolysis pathways

**Figure 2:**
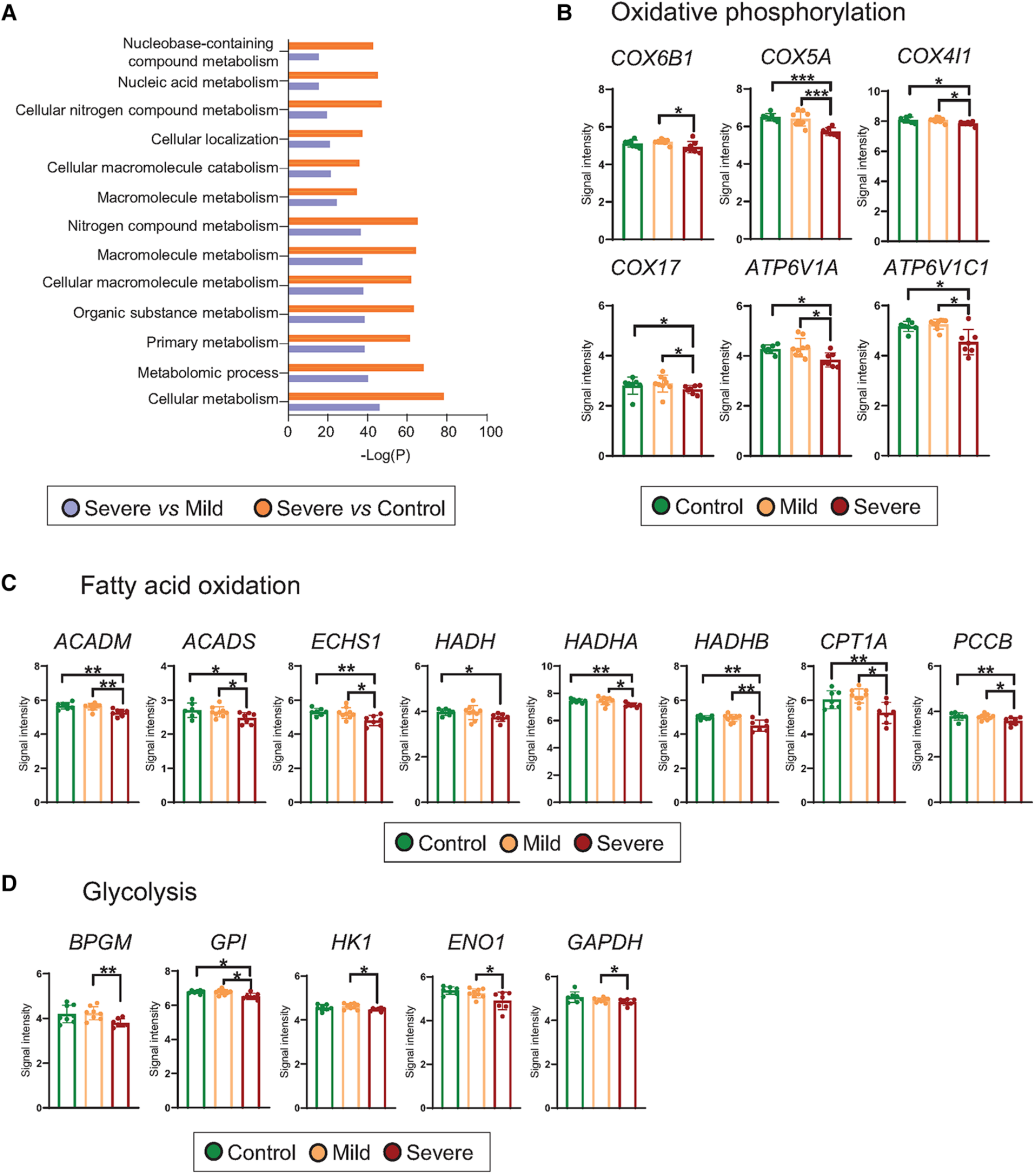
Metabolism of T cells from severe allergic patients is altered. (**A**) Top 10 differentially detected (*p* < 0.05) pathways using GOrilla Software in which DEGs from T cells of Severe vs. Control and Severe vs. Mild comparisons are involved. (**B–D**) Scatter dot plot representation of microarray intensities of genes involved in the specify metabolic routes, where mean + SD are shown: (**B**) oxidative phosphorylation (COX6B1, COX5A, COX4IL, COX17, ATP6V1A, ATP6V1C1), (**C**) fatty acid oxidation (ACADM, ACADS, ECHS1, HADH, HADHA, HADHB, CPT1A and PCCB and (**D**) glycolysis (BPGM, GPI, HK1, ENO1 and GAPDH). Plots show the mean + SD. *p < 0.05; **p < 0.01; ***p < 0.001.

In order to understand which cellular alterations were associated with the transcriptomic differences found between the experimental groups, we performed an enrichment pathway analysis (Supplementary Tables S4, S5). We identified that for both comparisons, severe *vs.* control and severe *vs.* mild, the most significant altered pathways were related to cellular metabolism, including macromolecular, primary, organic and nitrogen metabolism (Figure 2A and Supplementary Table S4). Although to a lower extent, mild-derived T cells exhibited changes related to RNA, regulation of FA metabolic and regulation of cell-cell adhesion processes in comparison to the control group (Supplementary Table S5). With the aim of identifying particular alterations, we deeply studied genes related to primary metabolic cellular pathways including OXPHOS, FAO, and glycolysis. Our results showed that severe phenotypes presented a downregulated expression in different cytochrome c oxidase subunits (COX), such as *COX6B1, COX5A, COX4IL and COX17,* concomitant with reduced levels of vacuolar ATPases transcripts, including *ATP6V1A* and *ATP6V1C*, all involved in OXPHOS (Figure 2B). Moreover, severe patients presented a downregulated expression of genes related to FAO, among them, Acyl-CoA Dehydrogenase Medium Chain (*ACADM*), Acyl-CoA Dehydrogenase Short Chain (*ACADS)*, Enoyl-CoA hydratase, short chain 1 (*ECHS1*), Hydroxyacyl-CoA Dehydrogenase (*HADH)*, Hydroxyacyl-CoA dehydrogenase trifunctional multienzyme complex subunit alpha (*HADHA)*, Hydroxyacyl-CoA dehydrogenase trifunctional multienzyme complex subunit beta (*HADHB)* and Propionyl-CoA Carboxylase Subunit Beta (*PCCB)*. We could also observe that the key-limiting FAO enzyme, carnitine palmitoyltransferase 1A *(CPT1A)* transcript, was significantly reduced in T cells from severe patients (Figure 2C). In addition, severe allergic asthmatic patients presented a downregulated expression of genes required for glycolysis such as Bisphosphoglycerate mutase (*BPGM)*, Glucose-6-Phosphate Isomerase (*GPI)*, Hexokinase 1 (*HK1)*, Enolase 1 (*ENO1)*, Glyceraldehyde-3-Phosphate Dehydrogenase *(GAPDH)* (Figure 2D). These findings were validated by qRT-PCR of selected differentially gene transcripts, including key genes for each metabolic route (Supplementary Figure S4). Finally, we evaluated mRNA expression levels of receptors linked to lipid signalling pathways and found that severe asthmatic patientśCD3 expressed significantly lower levels of genes related to G-protein-coupled receptors (GPR) such as *S1PR2 (sphingosine-1-phosphate receptor 2)*, *CYSLTR1* (Cysteinyl Leukotriene Receptor 1)*,* and different *GPR (E.g., GPR-19, 132 and −153)* in comparison to control individuaĺs CD3 (Supplementary Table S3 and Supplementary Figure S5), and lower levels of *S1PR2, S1PR5, and some GPR* (*E.g., GPR-108, 139* and *GPR180*) than mild subject´s CD3 (Supplementary Table S3 and Supplementary Figure S5). Altogether, our results suggest that T cells from severe allergic asthmatic patients present a decrease in genes related to metabolism in a threefold way, OXPHOS, FAO and glycolysis that might be link to significant metabolic changes.

### T cells from severe allergic asthmatic patients show altered immune activation and regulatory gene sets

**Figure 3:**
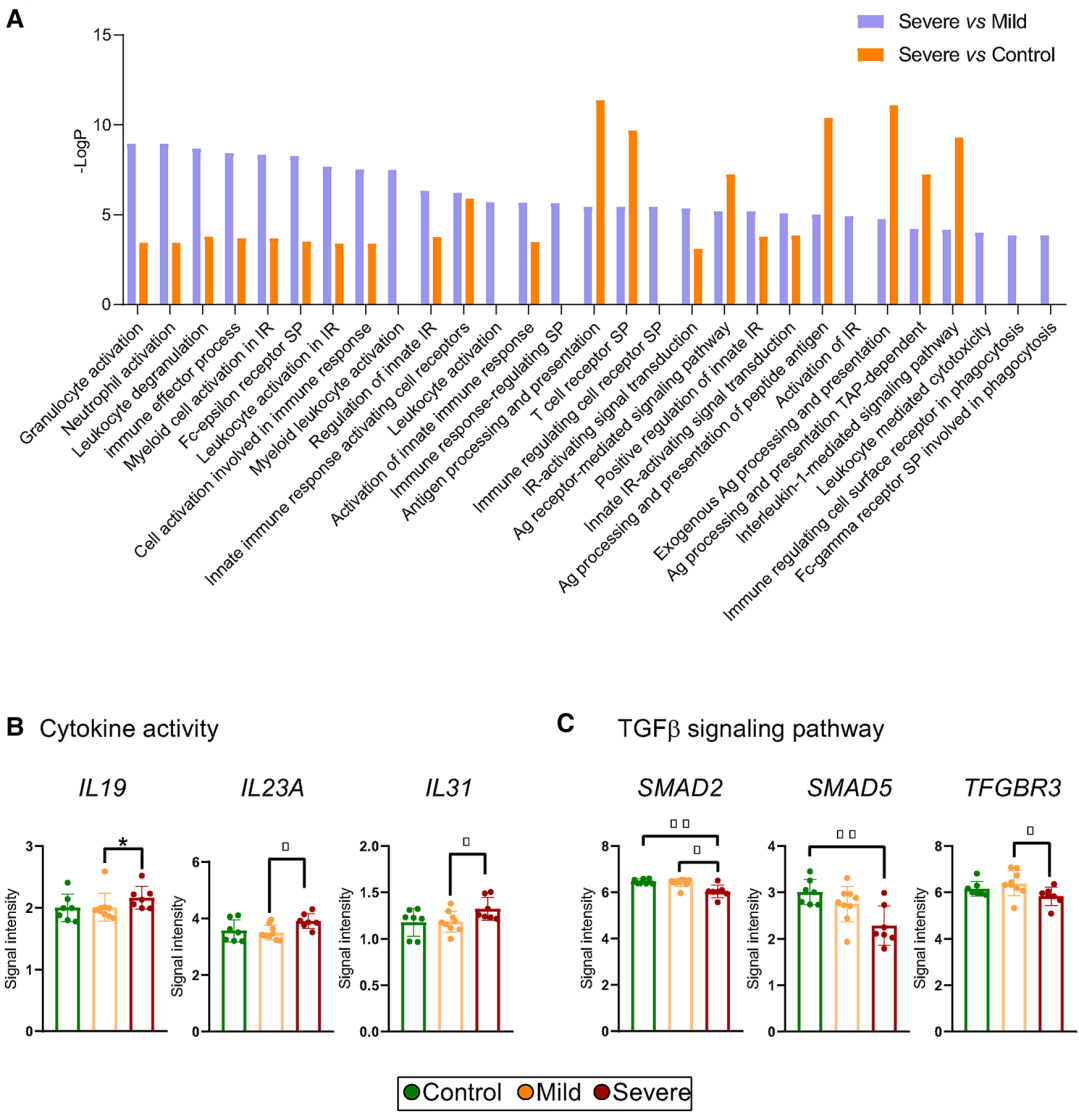
Immune activation pathways, cytokine production and TGF-*β* pathway alteration points to compromised treg cell function in severe allergic phenotypes. (**A**) Differentially detected (*p* < 0.05) pathways related to the immune response using GOrilla Software in which DEGs from T cells of Severe vs. Control and Severe vs. Mild comparisons are involved. (**B,C**) Scatter dot plot representation of microarray intensities of genes involved in (**B**) cytokine activity (IL-19, IL-23A and IL-31) and (**C**) in TGF*β* signalling pathway (SMAD5, SMAD2, TFGBR3). Plots show the mean + SD. *p < 0.05; **p < 0.01; ***p < 0.001.

The enrichment analysis pointed to modifications in pathways associated with the immune response. Interestingly, processes related to leukocyte activation and immune regulation were significantly boosted in both severe vs. mild and severe vs. control comparisons (Figure 3A). Accordingly, we studied genes related to cytokine production, observing that severe patients presented increased levels of pro-inflammatory-related transcripts such as *IL19, IL23A* and *IL31* (Figure 3B, Supplementary Table S3). Moreover, we investigated transcript expression levels of genes related to Treg cell functions. We found that severe allergic asthmatic patients presented modifications in mRNA levels of the TGF*β* Receptor 3 *(TFGBR3)* and the *SMAD* (mothers against decapentaplegic homolog) family of transcription factors, including *SMAD2* and *SMAD5* gene transcripts (Figure 3C). Since our data pointed to an alteration of TGF*β* signalling pathway of severe allergic asthmatic patients' T cells, we evaluated if Treg cell number was altered in these patients. Notably, we found that control subjects presented nearly twice as many Treg (CD4 + CD25+) cells compared with allergic patients expressed in relation to CD4+ T cells isolated by magnetic separation (control: 2,1 ± 51 0,7%, mild: 1,2 ± 0,3%, severe: 1,2 ± 0,4%), although not statistically significant (Supplementary Figure S6). In contrast, the number of Teff (CD4 + CD25-) cells appeared similar between the groups. Due to the isolation method used, additional technics could improve the accuracy of Treg and Teff cell number determination. Overall, our results suggest an imbalance in pro- and anti-inflammatory T cell functions, entailing an impairment in the regulatory response in severe allergic patients.

## Discussion

There has been a gradual rise in the occurrence of allergic disorders over the past few decades (14). Severe allergic asthmatic patients pose a clinical challenge in this context. These patients are not controlled with high doses of corticosteroids or biological drugs and as a consequence, have frequent excessive morbidity impacting the healthcare system and their quality of life (15, 16). Mechanisms underlying severe inflammation are poorly understood.

We have previously described that plasma samples from severe allergic patients presented a decrease in carbohydrates and pyruvate and an increase in lactate, implying Warburg metabolism (6). T lymphocytes possess an exceptional ability to alter their metabolism in response to both extracellular and intracellular signals, indicating their high degree of plasticity (9). It has been demonstrated that extracellular lactate and acidic conditions inhibit glycolysis in human T cells (17). Now, we show that T cells from severe allergic asthmatic patients present a downregulation of genes involved in glycolysis, which is in line with the above-mentioned findings. Our previous studies in PBMCs identified reduced OXPHOS, fatty acid metabolism, and adipogenesis in severe allergic patients (6). Moreover, persistent antigenic stimulation impairs OXPHOS, suppresses proliferation and upregulates genes linked to T cell exhaustion (18). This may explain the downregulation of genes involved in OXPHOS that we observed in severe allergic phenotypes. In fact, inhibition of OXPHOS has been observed in other inflammatory conditions such as sepsis and chronic human immunodeficiency virus infection (HIV) (19, 20). Furthermore, T cells from severe patients displayed decreased expression in genes related to FAO (21, 22), such as the rate-limiting enzyme *CPT1A*, which suggest reduced Treg cell generation. Interestingly, pathway analysis revealed that T cells from severe allergic asthmatic patients presented alterations in TGF*β* signaling pathway, which is critical to suppresses immune responses through the inhibition of inflammatory cells and the promotion of Treg function (23). This finding, together with the alterations observed in FAO, point to an altered regulatory function in severe allergic asthmatic patients. In fact, the lack of regulation capacity might be a key mechanism in the maintenance of the inflammatory state in these patients (1, 7, 24). We have already demonstrated that severe respiratory allergic patients presented an enhanced inflammatory immune response in the oral mucosa, with an increased number of CD4^+^ infiltrates (24) and enhanced recruitment of Treg cells (2). Also, we showed that uncontrolled asthmatic patients display altered levels of metabolites and proteins linked to inflammation and T cell activation (1). Taking everything into consideration, our data suggest an impaired regulatory function in severe allergic asthma.

In addition to metabolic changes, we confirmed that severe allergic asthmatic patients presented alterations related to immune activation. Of note, T cells from severe patients presented higher levels of *IL19, IL23A* and *IL31* transcripts. Concordantly, these three cytokines have been previously found increased in serum from asthmatic patients (25–27), and IL-31 plasma levels correlated positively with severity and Th2 related cytokines (27). Thus, our transcriptomic data indicate that T cells from severe patients could be a source of inflammatory cytokines such as IL-19, IL-23A and IL-31 that might contribute to maintain a chronic inflammatory status.

Altogether, our data provide significant information on the specific T-cell transcriptomic profile associated with severe asthmatic allergy. Moreover, this profile is linked to metabolic shift of T cells from severe allergic asthmatic patients, and points to an impaired regulatory function. Nevertheless, further *in vitro* validations need to be done. Understanding the mechanisms that drive the metabolic reprogramming of immune cells could be crucial in comprehending the progression towards severe allergic phenotypes and identifying novel biomarkers.

## Data Availability

The datasets presented in this study can be found in online repositories. The names of the repository/repositories and accession number(s) can be found in the article/**Supplementary Material**.
