# Supplementary material for: Transcriptomics reveals a distinct metabolic profile in T cells from severe allergic asthmatic patients
Source: Front Allergy. 2023 May 31;4:1129248. doi: 10.3389/falgy.2023.1129248 (PMC10265992; doi:10.3389/falgy.2023.1129248)
Supplement: Supplementary file 3 [file Presentation1.pdf]

Supplementary Material

Figure S1. Schematic representation of sample collection process.

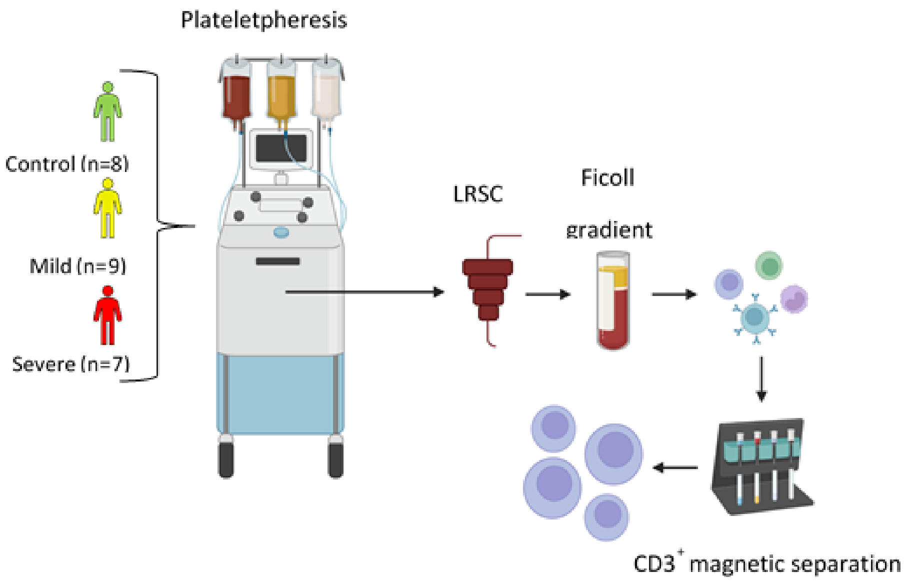

Figure S2. PLS-DA of LC-MS-positive mode showing differences between Control (n=8)*, Mild (n=9) and Severe (n=7)* of T cells' transcriptomic data. X axis indicates sample injection order in each group. One component was displayed and is indicated by Y axis.

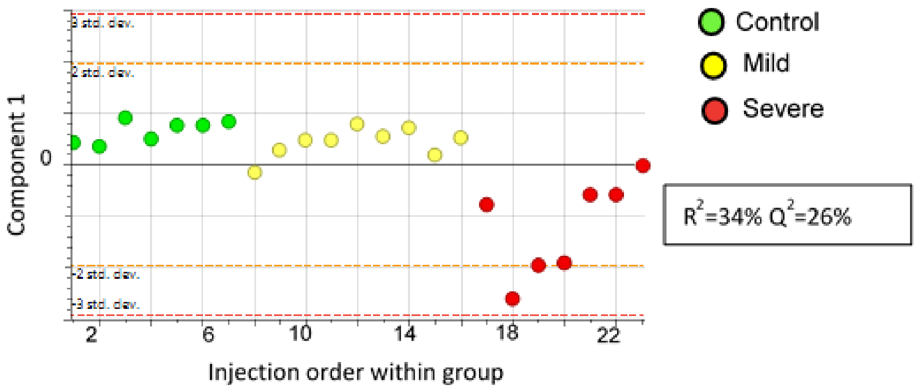

Figure S3. Transcriptomic alteration on CD3-positive cells isolated from mild and severe allergic asthmatic patients. (A) Differential analysis of CD3^{+} cells transcriptomic data from each comparison: Mild *vs.* Control (left) Severe *vs.* Control (centre) and Severe *vs.* Mild (right) shown as a volcano plot. X axis represents Log2(Fold change) and Y axis represents -Log10(P-value). Non-significative genes (Mann-Whitney p-value >0.05) are represented in gray dots. Statistically significant genes (Mann-Whitney p-value < 0.05) are represented in blue dots. Genes that showed a Log2(Fold change) greater than 0.5 and a p-value lower than 0.05 are represented in red dots. Selected genes that were subsequently validated by PCR are labeled in each volcano plot (*HLADRB5* and *HLADRB1* for mild *vs*. control, *SMS* and *NAMPT* for severe *vs.* mild and *HLADRB5*, *HLADRB1* and *NAMPT* for severe *vs*. control). **(B)** Hierarchical clustering was performed with the top 100-fold change DEGs between Mild *vs.* Control comparison. Each column represents a single transcript; each row represents an individual T cell sample. Red bands indicate a higher expression level, and blue bands indicate a lower expression level.

A)

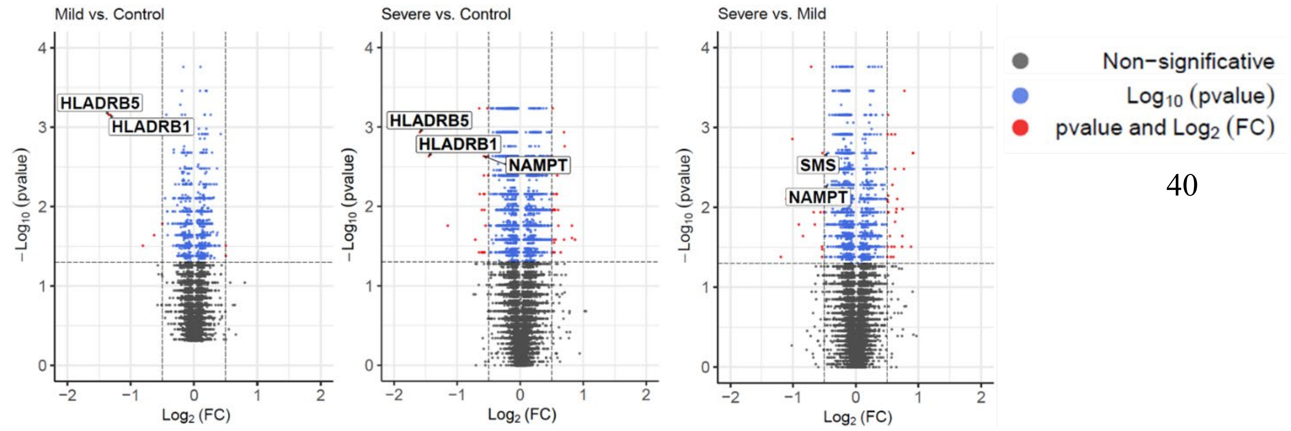

B)

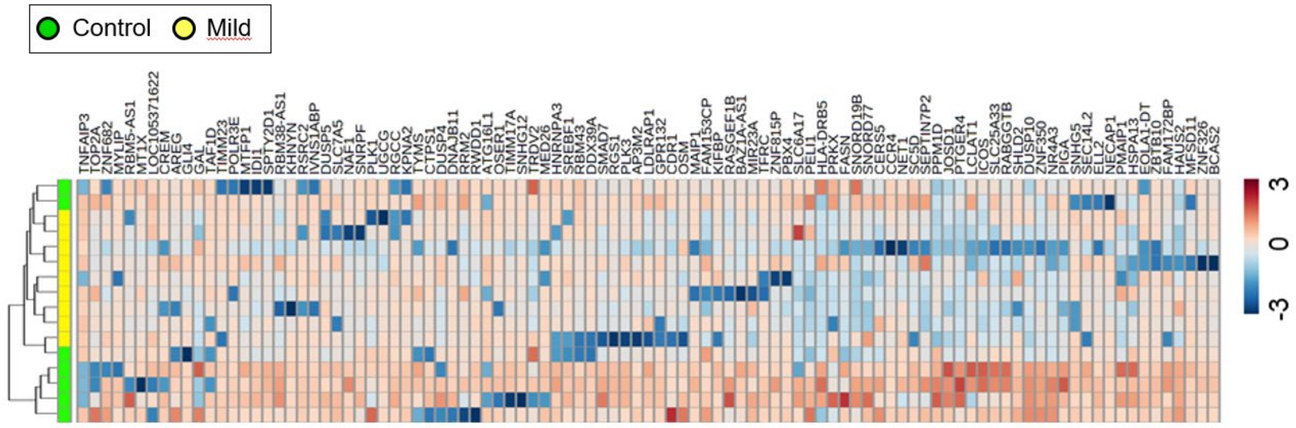

Figure S4. Quantitative real-time PCR validation of microarray findings in control subjects and mild and severe allergic patients for representative genes of different metabolic routes (Glyceraldehyde-3-phosphate dehydrogenase, *GAPDH*; carnitine palmitoyltransferase 1, *CTP1A;* and Cytochrome C Oxidase Subunit 5, *COX5*) and additional DEGs (Human Leukocyte Antigen DR5; *HLADR5B,* Human Leukocyte Antigen DR1, *HLADRB1;* Nicotinamide phosphoribosyltransferase*, NAMT;* and Spermine Synthase, *SMS*).

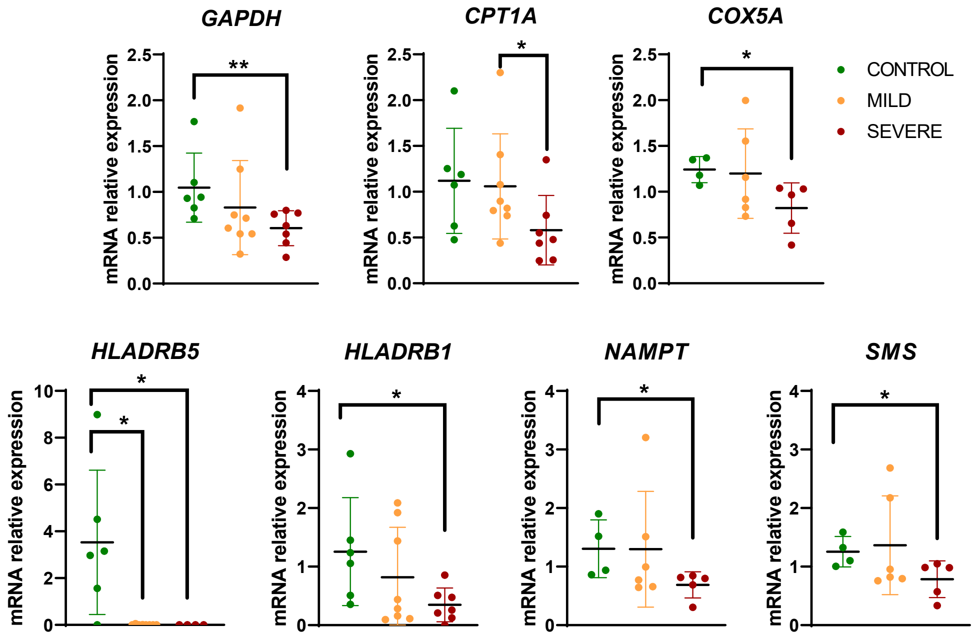

RNA from lysed CD3 cells was extracted using RNeasy Mini Kit (Qiagen) columns and later retrotranscribed into cDNA with the High Capacity RNA-to-cDNA kit (Applied Biosystems). SYBR Green master mix (Takara) was used for quantitative RT-PCR in the equipment CFX96 Real time system (Thermal Cycler C1000; Bio-Rad). We used *18S rRNA* as housekeeping gen. The ΔCt for all control samples was averaged to get ΔΔCt values for every sample (including for each control subject sample). Here we showed scatter dot plots with the fold change expression values ($2^{-(\Delta\Delta Ct)}$), where each dot represents a subject. Mean ± SD is shown. Statistical significance was assessed pairwise by Mann–Whitney test. *$P \leq 0.05$; **$P \leq 0.01$

Figure S5: Scatter dot plot representation of microarray intensities of selected DEGs associated to lipid signaling receptors that are differentially expressed on severe asthmatic allergic patients in comparison to control and mild group. Mean +SD are shown. Statistical significance was assessed pairwise by Mann–Whitney test. *$P \leq 0.05$; **$P \leq 0.01$. (*S1PR2,* sphingosine-1-phosphate receptor 2; GPR, G-protein-coupled receptors; *CYSLTR1,* Cysteinyl Leukotriene Receptor 1; *S1PR5,* sphingosine-1-phosphate receptor 5).

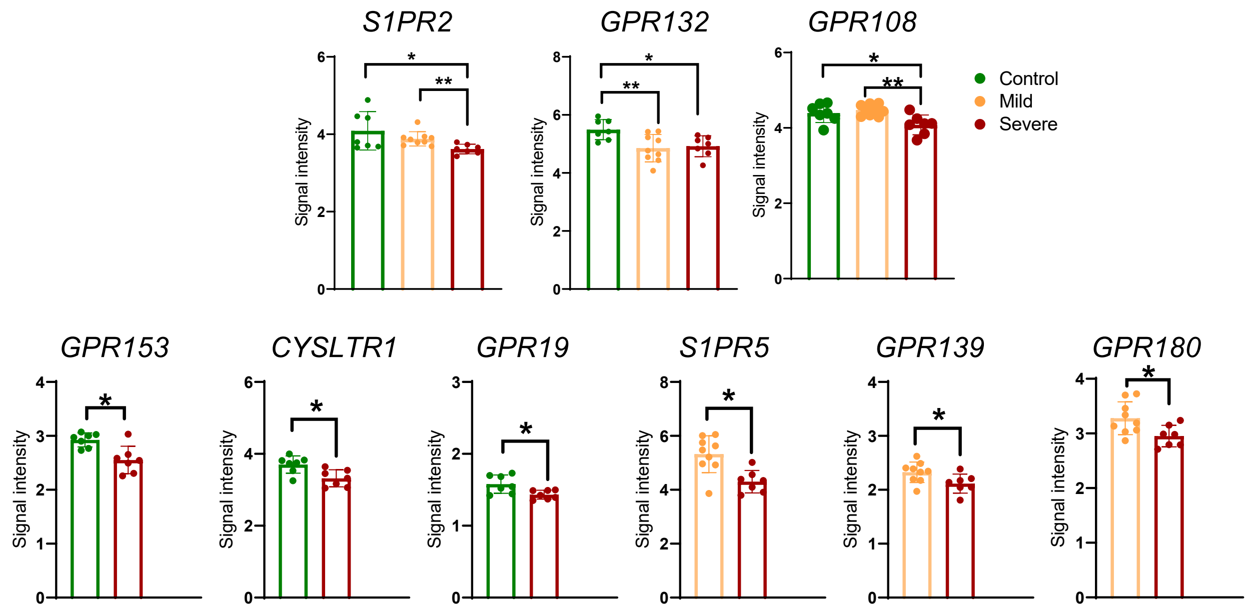

Figure S6. Percentage of Teff (CD4+CD25-) and Treg (CD4+CD25+) from the total of PBMCs in control (n=6), mild (n=3) and severe patients (n=6).

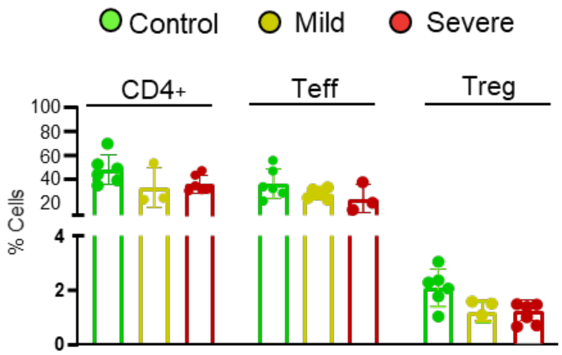

Table S1. Individualized subjects' information

Demographc information				Whole blood Hemogram (x10^9/L)
**Control**				
**Subject**	**Sex**	**Age**	**Smoking**	**WBC**
PL-08	F	26	no	7.98
PL-09	F	35	no	5.09
PL-10	M	38	yes	5.85
PL-11	F	26	no	5.77
PL-13	F	30	no	5.14
PL-24	F	41	no	7.01
PL-27	F	26	no	7.39
PL-12	F	27	no	4.57
**Mild**				
PL-02	F	37	no	6.23
PL-03	M	43	no	7.63
PL-04	F	35	yes	6.38
PL-05	F	27	no	6.54
PL-07	F	32	yes	6.95
PL-16	F	28	no	5.06
PL-22	F	22	no	8.18
PL-23	M	50	no	10.79
PL-25	F	26	no	7.86
**Severe**				
PL-14	F	53	no	6.35
PL-15	F	46	no	4.27
PL-17	F	28	no	7.06
PL-18	F	40	no	6.55
PL-20	F	21	no	5.70
PL-21	F	27	no	7.38
PL-26	F	36	no	7.63

Notes: F: Female. **M**: Male. **WBC**: White Blood Cells.

Table S2. Individualized patients' clinical information

					SPT (mm)												
**Patient**	**Onset age**	**Reactions**	**FVC (%)**	**FEV1 (%)**	**Ole**	**Phl**	**Cup**	**Pla**	**Cyn**	**Weeds**	**Fra**	**Pro**	**Alt**	**Dpt**	**Dfar**	**Cat**	**Dog**
Mild																	
PL-02	28	RC, AS	na	na	5x5	4x4	5x5	4x4			6x6						
PL-03	36	RC, AS	nv	nv	7x7	10x8	8x7	6x6		7x7	10x8						
PL-04	7	RC	na	na	7x6	4x4	5x5	4x4		8x4	4x4					6x6	
PL-05	15	RC, AS	nv	nv	5x5	5x6	5x5				15x9				7x5	10x10	
PL-07	13	RC, AS	nv	nv	6x3	12x6	14x12	3x3	9x9	3x3	5x5					7x5	
PL-16	10	RC, AS	nv	nv	4x4	9x4		5x5	8x4	5x4	6x4						
PL-22	15	RC, AS	nv	nv	9x8	6x6			6x5	6x6	8x7	5x4	6x6			6x7	3x3
PL-23	14	RC, AS	na	na	5x5	10x8	12x12	10x8	24x18	3x3		8x8	14x8				
PL-25	10	RC	na	na	6x7	5x6			11x6		6x8			5x4	6x6		
Severe																	
PL-14	23	RC, AS	nv	nv	10x8	17x12	8x7	10x9	7x7	4x4	8x7			3x3	3x3	6x5	9x9
PL-15	20	RC, AS	nv	nv	11x12	11x12	9x9		7x6	4x4				6x7	6x7	8x6	11x12
PL-17	15	RC, AS	62	60			5x5							8x5	7x7		
PL-18	27	RC, AS	69	65		10x11	7x7		4x4								
PL-20	6	RC, AS	68	53										6x6	11x9	12x8	9x9
PL-21	8	RC, AS	72	64			4x4					4x3					7x5
PL-26	22	RC, AS	69	73	4x6	5x4	4x3										

Notes: RC: Rhinoconjunctivitis**. AS:** Asthma**. FVC:** Forced Vital Capacity**. FEV1:** Forced Expiratory Volume in 1 second**. nv:** Normal Values**. na:** Not Available**. SPT:** Skin Prick Test**. Ole:** Olive**. Phleum:** Grass**. Cup:** Cupressus. **Pla:** Platanus. **Cyn:** Cynodon**. Fra:** Fraxinus. **Pro:** Profilin. **Alt:** Alternaria. **Dpt:** Dermatophagoides pteronyssinus. **Dfar:** Dermatophagoides farinae.

Table S5. Enrichment pathway analysis performed with DEGs in Control *vs.* Mild comparison by using GOrilla software

GO term	Description	P-value	FDR q-value	Enrichment (N, B, n, b)
Process (top 10)				
GO:0006396	RNA processing	1.88E-5	2.88E-1	1.13 (17561,1021,8387,552)
GO:0006139	nucleobase-containing compound metabolic process	3.28E-5	2.51E-1	1.07 (17561,2681,8387,1376)
GO:0016070	RNA metabolic process	3.42E-5	1.75E-1	1.10 (17561,1627,8387,854)
GO:0090304	nucleic acid metabolic process	3.44E-5	1.32E-1	1.08 (17561,2195,8387,1136)
GO:0046483	heterocycle metabolic process	6.13E-5	1.88E-1	1.07 (17561,2841,8387,1451)
GO:0019217	regulation of fatty acid metabolic process	6.61E-5	1.69E-1	1.43 (17561,91,8387,62)
GO:0006725	cellular aromatic compound metabolic process	1.03E-4	2.25E-1	1.07 (17561,2878,8387,1466)
GO:0034641	cellular nitrogen compound metabolic process	1.03E-4	1.98E-1	1.06 (17561,3168,8387,1608)
GO:2000047	regulation of cell-cell adhesion mediated by cadherin	1.79E-4	3.06E-1	1.87 (17561,19,8387,17)
GO:0000226	microtubule cytoskeleton organization	.89E-4	2.9E-1	1.19 (17561,370,8387,211)
Immune process (all)				
GO:0042130	negative regulation of T cell proliferation	2.66E-4	3.14E-1	1.48 (17561,61,8387,43)
GO:0002683	negative regulation of immune system process	6.89E-4	4.8E-1	1.16 (17561,423,8387,235)
GO:0050672	negative regulation of lymphocyte proliferation	7.21E-4	4.81E-1	1.40 (17561,75,8387,50)
